# Predicting Age From Large-Scale Brain Networks: Evidence From the Cam-CAN Dataset Across the Lifespan

**DOI:** 10.1093/geroni/igaa057.1177

**Published:** 2020-12-16

**Authors:** Meghan Caulfield, Irene Kan, Evangelia Chrysikou

**Affiliations:** 1 Villanova University, Villanova, Pennsylvania, United States; 2 Drexel University, Philadelphia, Pennsylvania, United States

## Abstract

Changes in cognition observed in aging (e.g. a shift from prioritization of fluid cognition in young adulthood toward an emphasis on crystalized knowledge and semantic cognition in older adulthood) are believed to reflect alterations in neural connectivity in aging. Recent work specifically highlights how increased connectivity between executive control (EC) regions and default mode network (DMN) may underlie characteristic shifts in cognitive abilities between younger and older adults. However, the contribution of the salience network, which plays a crucial role in mediating the dynamic interplay between EC and DMN, is relatively overlooked. To extend previous work, we used a large cohort (N = 547) of participants from the Cam-CAN database (18-88 years old) to examine whether resting-state functional connectivity between EC and DMN can reliably predict participant age. We further examined how addition of the salience network impacts the hypothesized increased connectivity between EC and DMN as a result of aging. A series of multiple regression analyses using functional connectivity and age as variables revealed that connectivity between EC and DMN regions (specifically between dorsolateral and ventromedial prefrontal cortex and parietal regions, including the precuneus) accounted for a significant portion of age variability and that the inclusion of the salience network improved the models’ explanatory power. Follow-up analyses by age cohort further highlighted that these relationships dynamically change across the lifespan. We will discuss these findings in the context of default-executive coupling hypothesis for aging and propose avenues for future research in refinement of this model.

